# Supporting health behaviour change in chronic obstructive pulmonary disease with telephone health-mentoring: insights from a qualitative study

**DOI:** 10.1186/1471-2296-13-55

**Published:** 2012-06-13

**Authors:** Julia A E Walters, Helen Cameron-Tucker, Helen Courtney-Pratt, Mark Nelson, Andrew Robinson, Jenn Scott, Paul Turner, E Haydn Walters, Richard Wood-Baker

**Affiliations:** 1 University of Tasmania, MS1 UTAS, Private Bag 23, Hobart, TAS, 7000, Australia; 2 University of Tasmania, Private Bag 135, Hobart, TAS, 7000, Australia; 3 Menzies Research Institute Tasmania, University of Tasmania, MS1 UTAS, Private Bag 23, Hobart, TAS, 7000, Australia; 4 University of Tasmania, Private Bag 30, Hobart, TAS, 7000, Australia; 5 University of Tasmania, Private Bag 100, Hobart, TAS, 7000, Australia

**Keywords:** Health-mentoring, Behaviour change, Primary care, Chronic obstructive pulmonary disease, Physical activity, Smoking

## Abstract

**Background:**

Adoption and maintenance of healthy behaviours is pivotal to chronic disease self-management as this influences disease progression and impact. This qualitative study investigated health behaviour changes adopted by participants with moderate or severe chronic obstructive pulmonary disease (COPD) recruited to a randomised controlled study of telephone-delivered health-mentoring.

**Methods:**

Community nurses trained as health-mentors used a patient-centred approach with COPD patients recruited in general practice to facilitate behaviour change, using a framework of health behaviours; ‘SNAPPS’ Smoking, Nutrition, Alcohol, Physical activity, Psychosocial well-being, and Symptom management, through regular phone calls over 12 months. Semi-structured interviews in a purposive sample sought feedback on mentoring and behaviour changes adopted. Interviews were analysed using iterative thematic and interpretative content approaches by two investigators.

**Results:**

Of 90 participants allocated to health-mentoring, 65 (72%) were invited for interview at 12-month follow up. The 44 interviewees, 75% with moderate COPD, had a median of 13 mentor contacts over 12 months, range 5–20. Interviewed participants (n = 44, 55% male, 43% current smokers, 75% moderate COPD) were representative of the total group with a mean age 65 years while 82% had at least one additional co-morbid chronic condition. Telephone delivery was highly acceptable and enabled good rapport. Participants rated ‘being listened to by a caring health professional’ as very valuable. Three participant groups were identified by attitude to health behaviour change: 14 (32%) actively making changes; 18 (41%) open to and making some changes and 12 (27%) more resistant to change. COPD severity or current smoking status was not related to group category. Mentoring increased awareness of COPD effects, helping develop and personalise behaviour change strategies, even by those not actively making changes. Physical activity was targeted by 43 (98%) participants and smoking by 14 (74%) current smokers with 21% reporting quitting. Motivation to maintain changes was increased by mentor support.

**Conclusions:**

Telephone delivery of health-mentoring is feasible and acceptable to people with COPD in primary care. Health behaviours targeted by this population, mostly with moderate disease, were mainly physical activity and smoking reduction or cessation. Health-mentoring increased motivation and assisted people to develop strategies for making and sustaining beneficial change.

**Trial registration:**

ACTR12608000112368

## Background

Chronic Obstructive Pulmonary Disease (COPD), a major cause of morbidity worldwide [1] is a common chronic condition seen in Australian general practice. The prevalence is 13.5% in primary care [2], with management occurring at a rate of 8 per 1000 general practitioner (GP) encounters [3]. Despite a high rate of misdiagnosis (30%) and delayed diagnosis [4,5], COPD management is often reactive [6] and COPD seen as subsidiary to the patients’ other chronic conditions [6,7]. However, the financial cost of COPD is very high for the health system and there is a large impact on individuals, with loss of productive healthy years of life [8]. A number of management approaches alongside conventional medical interventions have been deployed to try to reduce the COPD disease burden, such as patient-directed self-management interventions [9] including education or behavioural support [10].

Interventions to support self-management, [9,11] including adoption of health behaviours meeting guidelines for good health [12], could reduce the impact and slow deterioration in COPD. However, patients often have poor understanding of the nature of the disease and frequently fail to monitor symptoms [13] or make lifestyle changes that could have positive benefits [14]. They may experience marked impairment of quality of life impacting on activity, symptoms and participation in daily life [15]. They may also report poor knowledge about how to prevent worsening [16] and express confusion and even denial about smoking as the main causal factor [17].

In addition to smoking there are high rates of other preventable risk factors for disease in the Australian population, [18] including risky alcohol consumption, poor nutrition, and physical inactivity, which are targets for improvement highlighted in the recent national preventive health strategy [19]. However, there are challenges to achieving behaviour change around risk factors to improve long-term outcomes in COPD [20]. Without assistance to develop strategies for change, patient education and knowledge alone are not effective in improving health status [10][21]. This raises issues over the traditional approach to lifestyle counseling in primary care which is didactic rather than patient-oriented [22].

Self-management support interventions may be designed to address one or two specific health behaviours through education and coaching. Previous studies have addressed physical activity and diet in type 2 diabetes, hypertension in a primary care population [23] and seven specific coronary risk factors in established cardiac disease [24]. However, as the majority of patients with chronic disease in primary care have multiple co-morbidities, each with its own management priorities, an individual may benefit from behaviour change for multiple risks factors [25] and little is known about health behaviours that patients with chronic conditions themselves chose to address.

The qualitative study reported in this paper used interviews to investigate health behaviour changes adopted by people with moderate or severe COPD during a randomised controlled study of telephone-delivered health-mentoring to support self-management in primary care.

## Methods

### Selection of participants for interview

Participants were recruited from the telephone-delivered health-mentoring arm of a 12-month cluster randomised study undertaken in 31 general practices for patients with moderate or severe COPD. The study was approved by the Tasmanian Human Research Ethics Committee (H0009777) and was conducted between 2008 and 2010. Participants gave written informed consent and all those attending a 12-month follow up visit were invited to take part in the qualitative study. A purposive sample of those who agreed completed a semi-structured telephone interview of approximately 45 minutes duration. The sample selection sought to reflect a range of locations and number of health-mentor contacts, and study participants’ distribution by gender, smoking status, employment and educational level. Recruitment ceased after it appeared that the attributes of interviewees reflected our desired range and that we had sufficient to achieve content issue saturation.

### Health-mentoring

Nurses working in primary care as community or practice nurses were trained as health-mentors to assist people with chronic diseases to identify personal goals and health-related action plans directed to goal achievement. The two-day training programme was grounded in behavioural psychological theory and included: motivational interviewing skills; goal setting; action planning and problem solving; self-management support theory and practice; and a COPD-specific clinical management module [4]. Health-mentoring used a patient-centred approach to facilitate behaviour adoption or maintenance in the ‘SNAPPS’ framework of health behaviours; Smoking, Nutrition, Alcohol, Physical activity [26], Psychosocial well-being, and Symptom management. The intended mentoring schedule included 16 telephone calls, whose frequency varied from weekly initially then tapering to two monthly over 12 months.

### Data collection, outcomes and analysis

Participants’ demographic, anthropometric and co-morbidity data were collected and questionnaires were administered to assess symptoms and health status via the Medical Research Council (MRC) scale for functional dyspnoea [27], and the St George’s Respiratory Questionnaire (SGRQ) for health related quality of life at baseline.

Qualitative data were collected by a trained, experienced, independent researcher (KN) during a semi-structured interview completed within four weeks after 12-month follow up and sought feedback on the health-mentoring process and health behaviour adoption or maintenance. Interviews were audiotaped and transcribed verbatim and two researchers performed initial analysis using an iterative thematic approach [28]. After initial reading of transcripts, coding was performed independently by two researchers (JW and DW) who sought themes relating to health behaviour change and mentoring [29]. Emergent themes were subsequently discussed with other authors (HCT, HCP) and were examined using NVivo in relation to interviewees’ demographic status, disease severity and disease impacts. Interpretative content analysis was subsequently used to quantify interviewees’ health behaviour changes [28]. Findings are illustrated with quotes from participants whose age, smoking status and COPD severity at the start of the study are shown in parentheses.

## Results

Ninety study participants were allocated to the health-mentoring intervention, of whom 65 (72%) were invited for interview when attending 12-month follow up (11 withdrew before commencing the allocated intervention, 12 withdrew later without follow up, 2 died). The 44 interviewees, 33 with moderate COPD and with 11 severe COPD had a median of 13 mentor contacts over 12 months, range 5–20. Demographics, and disease impacts in the interviewees were comparable to the whole group of mentored participants, although the proportion of participants with COPD graded as moderate was greater in the interviewed (75%) compared to the non-interviewed (50%) participants (Table 1). COPD was the only chronic disease reported by 18% of interviewees, 39% had one other chronic disease and 34% had two or three other chronic conditions.

**Table 1:** Characteristics of interviewed participants (n = 44) and non-interviewed participants (n = 46) in a study of telephone delivered health mentoring in COPD

	**Interviewed n = 44**		**No interview n = 46**	
	**Mean**	**SD**	**Mean**	**SD**
Age (years)	65.2	7.3	64.3	8.5
Functional dyspnoea (MRC 1–5)^1^	2.6	1.1	2.9	1.3
Health related quality of life SGRQ (0–100 worst)^2^	41.3	21.1	42.6	18.4
	**n**	**%**	**n**	**%**
Current smoker	21	48	23	50
Male	24	55	24	52
COPD severity*				
FEV1 50–80% predicted = Moderate	33	75	23	50
FEV1 30–50% predicted = Severe	11	25	23	50
Living alone	18	41	18	39
Overweight or obese ^3^	30	68	29	64
Employed currently	11	25	8	17
Education level				
Primary	3	7	8	17
Year 7-10	25	57	24	52
Year 11 or 12	6	14	6	13
Certificate/diploma	6	14	5	11
University degree	4	9	3	7
Previous self-management education	3	7	6	13

Footnotes: ^1^ MRC = Medical Research Council scale; ^2^ SGRQ = St George’s Respiratory Questionnaire; ^3^ Overweight or obese = BMI ≥25 kg/m^2^. * Chi^2^ p < 0.05.

### Impact of health-mentoring on making health behaviour changes

The experience of participating in telephone health-mentoring was for most participants, a valuable one. They believed that mentors helped them to identify goals, activities and strategies that assisted them with management of their COPD and their general wellbeing. The majority of participants indicated a change in health behaviours as a consequence of their participation in the telephone mentoring with 14 (32%) actively making health behaviour changes, 18 (41%) showing openness to change and making some small change(s), and 12 (27%) appearing somewhat more resistant to making changes. Behaviour change responses were not related to the participants’ severity of COPD. Based on analysis of interview transcripts, the most frequent changes in health behaviours occurred in relation to physical activity (43 participants, 98%), reducing smoking (14 participants, 74% current smokers) or achieving smoking cessation (21% of current smokers reported having quit at interview), nutrition (10 participants, 23%) and symptom management (8 participants, 18%).

### Changing smoking behaviour

Participants who were still actively smoking during the course of the programme reported that mentoring had changed the way they thought about their smoking, which in turn led to changes in their smoking habits:

"“As I started saying it was the philosophy behind why one habitually does something, no matter what. The purpose behind it, the reason for it, etcetera, etcetera. And she [the mentor] always brought me back round [in the discussion] to where I was at that time. And every time she rang me I had taken a step. And it was really good to discuss with her. And she knew from the word go, because I made it quite clear, do not expect miracles… But you know, suddenly because, because our call was important… I’d find myself thinking, you know, suddenly something would strike me and oh, I will leave those [the packet of cigarettes] inside while I go and plough the field. And yeah, I’m not taking them in the car today…. So she made me very aware of what I was doing, instead of doing it automatically.” (#4045, female, age 62, smoker, moderate COPD)"

Another participant talked about how he had managed to quit smoking during the course of the program, and how the support of the mentor had helped achieve this when he said “*They were sort of complimenting me on what I was doing. Or trying to do”* (#4041, male, age 66, smoker, moderate COPD)

Mentor support was seen as different from, and more effective, than family advice:

"“[the mentor] somebody different to talk to, to open up to. Um, somebody more understanding ah, whereas at home all I cop is ‘just give the bloody things up’. You know, ‘it’s simple’ all that sort of thing. Yeah, it was easier to talk to [the mentor] than it is to talk to my wife about it… And the end result will be that I will give up the cigarettes. I haven’t done so yet, but I will. What I did do was reduce the number I was taking quite significantly.” (#4071, male, age 59, smoker, moderate COPD)"

The opportunity to discuss personal health issues with someone outside the family circle was valued:

"“Being able to talk to somebody outside the family about what was wrong with you. That’s always handy, because the family are a bit close and you know, they have to live with it, which makes it very difficult for them.” (#4049, female, age 64, smoker, severe COPD)"

Considering simple strategies with the health-mentor gave participants the knowledge they needed to make changes, “*She said ‘Well, why don’t you leave them at home?’ I said ‘Well I never thought of that!’*” (#4559, male, age 71, smoker, moderate COPD). Sometimes participants believed that they had been able to make changes to their smoking because of the strategies their mentor had provided to cut down or give up:

"“And on the smoking side, it [mentoring] was excellent. She [my mentor] gave me the ways and means of trying to either cut down [my smoking] dramatically, which I did do…” (#4063, female, age 68, smoker, moderate COPD)"

### Changing physical activity

A focus on exercise adoption or maintenance was also something that most participants discussed, and this health behaviour change was demonstrated through participation in this program.

"“… I suppose I was more aware of what I must do for my lungs. Like the exercising and that sort of thing. So that did make me focus a little bit on that [exercising], which was good because that is something that I can help… But she was a great moral support. And yes, I was able to sort of focus and to know what I needed to do for the lungs” (#4600, female, age 67, exsmoker, moderate COPD)"

The experience of participating in telephone health-mentoring made participants reassess some of the activities of daily living which they had taken for granted, such as exercise, which in turn led to behaviour change. One participant talked about how the health-mentoring had made them think about how to change their normal routines to include more exercise, leading to achieving more exercise by *“just giving it a try”* (#4041, male, age 66, smoker, moderate COPD*).*

"“Parking in say one spot, and going to the post office and bakery and the chemist from the one spot instead of going to the chemist and getting in the car and driving down to the bakery and the post office” (#4041, male, age 66, smoker, moderate COPD)"

Another participant indicated a similar sentiment:

"“I didn’t think about doing these things [increasing my exercise] … but she said ‘What about going for a walk?’.. and you know, [now] I have a walk every day kind of thing… once I started on it, it was alright” (#4559, male, age 71, smoker, moderate COPD)"

### Motivational elements of health-mentoring

Participants identified several ways that telephone health-mentoring supported them in making health behaviour changes. For some participants, health-mentoring provided the regular reminder they needed to instigate changes:

"“Most of the things… were pretty obvious. I just needed that little bit of a prompt to get me to do them sort of thing”. (#4556, female, age 65, exsmoker, moderate COPD)"

Participating in health-mentoring also helped to reinforce existing health behaviours by raising and refocusing participant awareness on the management of their COPD, and what positive health behaviours they could be doing, as distinct from just providing information:

"“No, not the knowledge, [it was] not the knowledge, but strategies, in terms of… just sort of being consciously aware [of the need to change my behaviours], rather than sort of automatically doing something. And [now] I’ll think to myself consciously… do this… do that. So she [my mentor] was instrumental in that” (#4045, female, age 62, smoker, moderate COPD)"

Another participant stated

"“It [the health-mentoring] made me go back to thinking about myself and watching [what I did]. You know, being aware of what I was doing” (#4600, female, age 67, exsmoker, moderate COPD)."

The experience of telephone health-mentoring created a partnership between the participant and the mentor, which encouraged participants to commence, strengthen or continue positive health behaviours. In some cases the participant felt that they had a responsibility to their health-mentor to try and achieve some of the goals they had discussed. Additionally, just the knowledge that the mentor would be calling was also sometimes enough for participants to make positive health choices or change health behaviours. For example, one participant stated:

"‘Well mentally to know that I am getting a phone call every now and again was a big help’ (#4088, female, age 68, exsmoker, moderate COPD)."

Another discussed the feeling of accountability she felt as a participant in the program and how this helped motivate her to increase her physical activity:

"“Oh basically it [working with the health-mentor] was just support and motivation. Like if I knew she was ringing I would think I better go and do a couple of walks, because she is going to bloody ask me” (#4510, female, age 60, exsmoker, severe COPD)"

Participants persisted with behaviour changes through health-mentors’ encouragement and fostering problem solving

"“Well when [the health-mentor] would ring again, I’d say to [the health-mentor]… ‘I didn’t quite get this’ and [the health-mentor] would say ‘well you know, think about types of things why and what sort of things and things like that’ and then I would go that step further and look that step further” (#4037, female, age 45, exsmoker, moderate COPD)"

Participants with severe COPD reported the need for perseverance to overcome difficulties in making behaviour changes.

"“Well I push myself all the time. I wont let it get on top of me, because it can be very easy to do that. Um, throw in the towel if you like… I go outside and I force myself to do things. I got to push my body along as much as I can. I still do quite a bit of work. It’s slow, but I still do it” (#4125, male, age 70, exsmoker, severe COPD)"

## Discussion

Telephone health-mentoring delivered by trained nurses working in a community setting was favourably received by people with moderate or severe COPD, of whom the majority had other co-morbid chronic conditions. They reported that it assisted them to focus on and modify or adopt health behaviours. They most frequently targeted physical activity and stopping smoking. Health-mentoring increased mentees’ motivation, helped them to develop strategies for change and to sustain those behavioural changes.

Traditionally self-management support interventions, delivered by telephone, are targeted at only one or two specific health behaviours, such as physical activity in sedentary people at increased risk of type 2 diabetes [30], or physical activity and diet in type 2 diabetes and hypertension [31]. The target health behaviours are usually determined by the perceived importance to the health professional for disease causation in the population being studied. In clinical practice, primary care health professionals show considerable variation in assessing lifestyle behavioural risk factors [32], but again treatment choices are generally influenced by the health professionals’ beliefs and attitudes, and also by geographic location, consultation context and the patient’s socio-economic status. Surprisingly, there has been little published and there is little relevant information on the patient’s perspective on choice of target health behaviours. In contrast, health-mentoring delivers individual self-management support that is patient-focused. The health-mentor training given to community nurses in this study deliberately focused on a range of health behaviours linked to known risk factors for chronic diseases [26] but a key message taught was to facilitate individual participant choice.

Smoking is the principal risk factor for development of COPD and thus might be predicted as a target for behaviour change in people suffering from the condition. However, some smokers construct a narrative in which they do not accept smoking to be the main reason for development of their own COPD [17] and smoking prevalence is actually greater among people with more severe disease [33]. Of note in this study, a majority of smokers reported changes in smoking behaviour, and although reduction of smoking rather than cessation sometimes resulted, the attitude and motivation of most smokers demonstrated change. This is important because the study of Williams et al. [34] has shown that support to make a clear and autonomous decision about whether or not to make a quit attempt increases cessation in smokers not otherwise motivated to quit - such a decision was found to be a critical factor in enhancing their motivation.

The choice of physical activity as target for change by almost all participants reflects the impact of COPD and impaired functional capacity, compared to healthy elderly people [35]. This is important because increased physical activity is likely to be beneficial for individuals with COPD, improving their quality of life [36] and reducing the decline in their lung function [37].

Another study using a comprehensive face-to-face hospital based self-management education program in COPD [10] found that in addition to physical activity, improved nutrition and managing deteriorating symptoms were behaviours addressed by some patients. In that study participants had very severe COPD and behaviour change was reinforced with regular follow-up telephone calls, but only around a quarter still smoked [21]. In our study, participants were recruited in primary care with less severe COPD, nearly half being current smokers whose future course would benefit greatly from quitting.

Participants interviewed in our study were representative of the whole health-mentored group and reflected the typical primary care COPD population in Australia in having multiple chronic disease comorbidities [25]. Thus the results are likely to be generalisable to the whole COPD primary care population.

## Conclusions

Engaging people in health behaviour change is recognized as a high priority in primary care [19]. However, achieving and maintaining change is difficult. This may be especially true in COPD, with patients experiencing helplessness that is reinforced by repeated bad experiences [38]. The qualitative results from this study of health-mentoring have demonstrated that supporting participants’ autonomy in health behaviour adoption or maintenance was possible, and can be effective in increasing their self-motivation for change [39]. It was apparent in our study that health behaviours targeted for change were appropriate to prevention and improving quality of life in COPD while being directed by participant preference. This is encouraging for direct patient coaching telephone services that have recently become available in parts of Australia (https://www.gethealthynsw.com.au/) for prevention of chronic disease in adults at risk of developing chronic diseases from some lifestyle risk factors such as diet and inadequate physical activity. However a notable omission from the target current specified risk factors is smoking cessation, which was the choice for action by the majority of current smokers in this study.

## Abbreviations

COPD, Chronic Obstructive Pulmonary Disease; MRC, Medical Research Council; SD, Standard deviation; SGRQ, St George’s Respiratory Questionnaire; FEV1, Forced expiratory volume in 1 second.

## Competing interests

The authors declare that they have no competing interests.

## Authors’ contributions

JW undertook initial analysis of interview transcripts, and HCP and HCT participated in discussions about analyses of the study and the reporting. JW, HPC and HCT drafted the manuscript. HCT developed the health-mentor training. MN, AR, JS, PT, EHW and RWB were involved in all aspects of the study. All authors read and approved the final version of the manuscript.

## Pre-publication history

The pre-publication history for this paper can be accessed here:

http://www.biomedcentral.com/1471-2296/13/55/prepub
